# Association between zinc and chronic hepatitis: A 2-sample Mendelian randomization study

**DOI:** 10.1097/MD.0000000000044494

**Published:** 2026-01-23

**Authors:** ZhengHao Zhang, TianYang Chen, Yong Qi

**Affiliations:** a The First Clinical Medical College, Shandong University of Traditional Chinese Medicine, Jinan, China; b College of Traditional Chinese Medicine, Changchun University of Chinese Medicine, Changchun, China; c Department of Hepatology, Tai'an Hospital of Traditional Chinese Medicine, Taian, Shandong Province, China.

**Keywords:** causal research, chronic hepatitis, Mendelian randomization analysis, micronutrients, zinc

## Abstract

Micronutrients play a critical role in the development and progression of chronic hepatitis (CH). Variations in the levels of these essential nutrients can significantly influence disease outcomes in CH patients. This study investigates the causal relationships between 14 key micronutrients – copper, zinc, magnesium, vitamins A, C, D, B_6_, B_12_, folate, carotene, iron, selenium, calcium, and potassium – and the pathogenesis of CH. We utilized Mendelian randomization (MR) to explore causal relationships between 14 micronutrients and CH. Instrumental variables for micronutrient levels were derived from large-scale genome-wide association studies in European populations, with CH outcome data sourced from the FinnGen database. Our MR analysis employed 5 methodologies – inverse-variance weighting (primary approach), MR-Egger, weighted median, simple mode, and weighted mode – to identify micronutrients linked to CH. Sensitivity analyses assessed heterogeneity and pleiotropy, while a multivariable MR approach, supported by further sensitivity analyses, ensured the robustness of the findings. The inverse-variance weighting analysis revealed a significant causal association between zinc levels and CH, with a *P*-value of .022, an odds ratio of 0.806, and a 95% confidence interval of 0.670 to 0.970. Sensitivity analyses confirmed the robustness and reliability of this finding, with no evidence of heterogeneity or pleiotropy affecting the results. This study demonstrates a protective effect of zinc against CH, establishing a significant causal relationship. These findings provide foundational insights that advance our understanding of CH pathogenesis and support the development of targeted therapeutic interventions for its management.

## 1. Introduction

The liver, a vital organ responsible for detoxification, metabolism, and filtration, orchestrates numerous complex physiological processes essential to human health. Hepatitis, characterized by liver inflammation, arises from diverse etiologies, including viral infections, drugs, toxins, or autoimmune reactions. Clinically, hepatitis is classified based on duration, with persistent inflammation exceeding 6 months diagnosed as chronic hepatitis (CH). Histologically, CH is marked by portal lymphocyte infiltration, pronounced hepatic inflammation, hepatocyte injury, and liver fibrosis.^[1]^ The etiology of CH is multifaceted, encompassing viral infections (notably hepatitis B virus [HBV] and hepatitis C virus [HCV]), autoimmune disorders (e.g., autoimmune hepatitis and primary sclerosing cholangitis), drug-induced liver injury, metabolic disorders, and excessive alcohol consumption. Among these, HBV and HCV are the predominant contributors to CH. According to the World Health Organization, in 2019, the global prevalence of HBV infection was approximately 3.8%, with 1.5 million new infections annually. Of the 296 million individuals with chronic hepatitis B, 8,20,000 deaths were attributed to HBV-related liver diseases. Similarly, the prevalence of HCV infection was 0.8%, with 2,90,000 deaths from liver diseases among 58 million individuals with chronic hepatitis C (CHC).^[2]^ HBV and HCV replicate without directly damaging hepatocytes; however, during the chronic phase, inadequate T lymphocyte (T-cell) responses fail to eliminate these viruses from the liver, perpetuating a cycle of minimal cellular damage that progresses to CH.^[3]^ Despite differences in immune mechanisms driving HBV and HCV persistence, both exhibit diminished virus-specific T-cell responses in chronic stages, resulting in functional exhaustion of CD4 and CD8 T cells. This is compounded by inhibitory molecules, such as programmed cell death protein 1 and T-cell immunoglobulin and mucin-domain containing protein 3, which promote the secretion of immunosuppressive cytokines, including interleukin-10 and transforming growth factor-β. Additionally, natural killer cells exert regulatory effects that mitigate liver inflammation and delay disease progression.^[4]^ CH manifests as persistent liver disease characterized by ongoing hepatocyte regeneration and inflammation, which may culminate in hepatocellular carcinoma (HCC). A meta-analysis of 17,374 patients across 11 global prospective studies identified HCC as the leading cause of mortality in CH patients.^[5]^ Consequently, identifying modifiable risk factors for CH progression is paramount to inform preventive and therapeutic strategies.^[6]^

Micronutrients are essential for maintaining human health and supporting vital physiological processes.^[7]^ Iron, a critical micronutrient, constitutes approximately 5 grams in the human body, with roughly half incorporated into hemoglobin in red blood cells and most of the remainder bound to ferritin complexes in the bone marrow, liver, and spleen.^[8]^ Chronic viral hepatitis, particularly CHC, frequently disrupts iron metabolism, leading to iron overload in many patients. This is attributed to reduced hepcidin levels and elevated transferrin receptor 2 expression, which facilitate iron transfer from macrophage stores and intestinal mucosa to hepatocytes.^[9-11]^ Excessive iron accumulation promotes the generation of intracellular reactive oxygen species, inducing oxidative stress (OS) and potential liver damage.^[12,13]^ Vitamin D, an immunomodulatory micronutrient, mitigates inflammation by reducing pro-inflammatory cytokines and enhancing protective immune responses. In CHC patients, 1,25-dihydroxyvitamin D [1,25(OH)₂D] suppresses toll-like receptor expression, thereby decreasing the secretion of inflammatory mediators such as tumor necrosis factor-α and C-X-C motif chemokine ligand 10.^[14]^ Additionally, vitamin D inhibits inflammatory T-cell activity, reducing levels of interferon-gamma and interleukin-17.^[15,16]^ Studies indicate a prevalent vitamin D deficiency in patients with liver disorders, including chronic hepatitis B, CHC, alcoholic fatty liver disease (AFLD), and non-AFLD, partly due to chronic inflammation accelerating the conversion of 25-hydroxyvitamin D [25(OH)D] to 1,25(OH)₂D, thus shortening its half-life.^[17]^ Furthermore, alcoholic liver disease (ALD) is associated with micronutrient deficiencies, as alcohol provides calories but lacks essential micronutrients.^[18]^

Mendelian randomization (MR) analysis is an application that applies instrumental variable (IV), aim to assess causal relationships in non-experimental data.^[19]^ The aim is to identify genetic variants associated with the exposure. The genetic variant is required to be independent of confounding factors while not directly influencing the outcome. That is, the genetic variant is directly related to and only related to the exposure. There by guaranteeing that any relationship with the outcome is exclusively mediated by the exposure itself. In MR studies, single nucleotide polymorphisms (SNPs) linked to the exposure function as IV for evaluating causal relationships with the exposure and outcome. Common genetic variants are generally independent within populations and disassociated from confounding variables, thus circumventing the biases frequently encountered in traditional epidemiological methods.^[20]^ This research employs MR to examine the impact of 14 micronutrients – on CH. The study aims to elucidate the contribution of these micronutrients to CH progression and to inform improvements in clinical diagnostics and therapeutic approaches.^[21-23]^

## 2. Materials and methods

### 2.1. Study design

**Figure 1.**
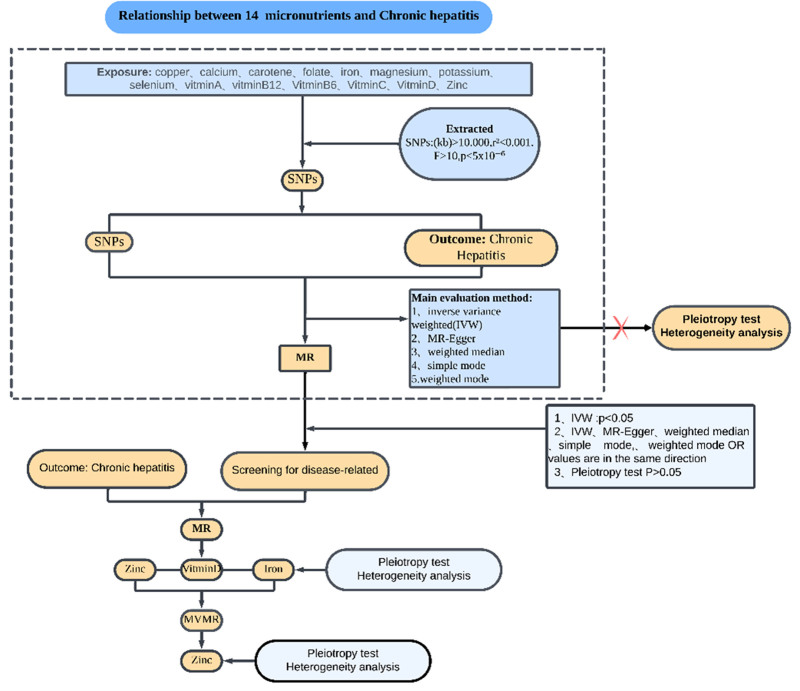
Summary of the MR study design for the relationship between 14 micronutrients and CH. CH = chronic hepatitis, IVW = inverse variance weighted, MR = Mendelian randomization, MVMR = multivariable Mendelian randomization, SNP = single nucleotide polymorphism.

**Figure 2.**
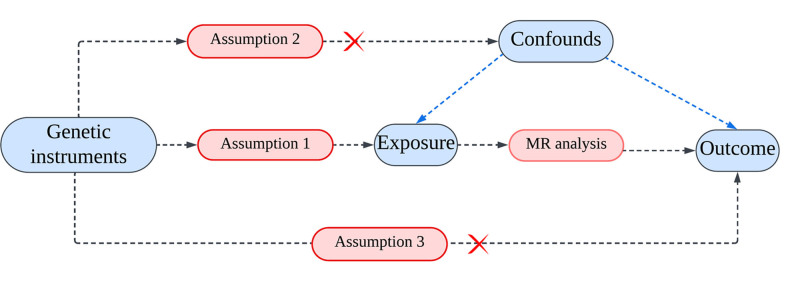
Hypothesis 1: the instrumental variable (SNP) is significantly associated with the exposure (micronutrients); hypothesis 2: the instrumental variable does not affect the outcome (CH) through confounding factors; hypothesis 3: the instrumental variable is not directly linked to the outcome but impacts it indirectly through its effect on the exposure. CH = chronic hepatitis, MR = Mendelian randomization, SNP = single nucleotide polymorphism.

This study employed MR, a method often analogized to a “natural randomized controlled trial.” In Figure 1, the research methodology is displayed, showcasing the acquisition of 14 exposure datasets from the OPEN GWAS database, alongside 1 outcome dataset related to genome-wide association studies (GWAS) obtained from the Finn Gen biobank. All datasets comprised individuals of European ancestry. Figure 2 depicts the compliance with 3 essential MR hypotheses: firstly, the genetic variants must be strongly associated with the exposure. Secondly, the instrumental variables should be independent of confounding factors; that is to say: IVs are to influence the outcome through exposure rather than confounders. Thirdly, the IV is linked to the outcome only indirectly through its effect on the exposure.

### 2.2. Data sources

**Figure 3.**
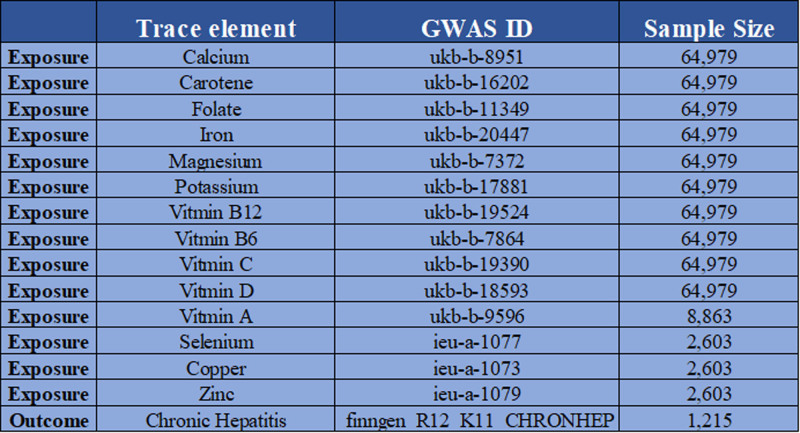
Exposure data from the Open GWAS database for calcium, carotene, folate, iron, magnesium, potassium, vitamins B_12_, B_6_, C, D, A, copper, and zinc. Exposure: CH. CH = chronic hepatitis, GWAS = genome-wide association studies.

The micronutrient GWAS data used in this investigation came from the Integrative Epidemiology Unit Open Genome-Wide Association Study (IEU Open GWAS) database. Figure 3 shows the 14 micronutrients’ summary data. The sample sizes of magnesium, potassium, calcium, carotene, folate, iron, vitamins B12, B6, C, D, data all contained 64,979 Europeans. Their GWAS IDs were ukb-b-7372, ukb-b-17881, ukb-b-8951, ukb-b-16202, ukb-b-11349, ukb-b-20447, ukb-b-19524, ukb-b-7864, ukb-b-19390, ukb-b-18593. The sample size for the vitamin A (ukb-b-9596) data contained 8863 Europeans, and the sample size for the selenium (ieu-a-1077), copper (ieu-a-1073), and zinc (ieu-a-1079) data all contained 2603 Europeans. Figure 3 shows that the outcome data (dataset ID: finngen_R12_K11_CHRONHEP) were provided by Finn Gen and included 1215 CH patients and 4,85,213 controls. On the Finn Gen website, you can find detailed procedures about quality standards, genetic analysis, and imputation. Ethical approval and informed permission were not needed because these data are from publicly available sources; this ensures that the data is transparent and that the results can be relied upon for wider medical research dissemination.

### 2.3. Selection of instrumental variable

We developed precise selection criteria to identify SNPs associated with the exposure. A significance level of 5 × 10^−6^ was established for the entire genome. The clumping parameters were set at *r*^2^ < 0.001 and kb = 10,000 in order to guarantee that SNPs were independent and to remove those that were in linkage disequilibrium. This allowed us to select SNPs that were associated with the exposure. From a statistical perspective, a weak instrument refers to a variable that has a weak association with the exposure. However, weak instruments still satisfy tvhe IV assumptions, making them valid instruments. Despite the asymptotic unbiasedness of weak instruments, which suggests biases diminish as sample sizes increase, finite sample sizes can still yield biased estimates. Here is the formula we used to get the *F*-statistic for each SNP: *F* = *R*^2^ (N − *K* − 1)/*K* (1 − *R*^2^), where *R*^2^ is the variation in the exposure explained by the SNP, N is the sample size, and *K* is the number of instrumental factors. We did this to assess the strength of the relationship between the SNP and the exposure. Exclusion from the analysis was granted to SNPs having an *F*-statistic below 10. Using this method, we can be sure that the SNPs we choose as instrumental factors for the MR analysis will have high relationships with the exposure.

### 2.4. Statistical analysis

This investigation utilized R (version 4.42) and the Two Sample MR package for all analyses. While MR-Egger, weighted median, basic mode, and weighted mode were all useful, the inverse-variance weighting (IVW) approach was by far the most popular.^[24]^ Originating from meta-analysis concepts, IVW minimizes variance through weighted averages, establishing it as a widely accepted and dependable method.^[25]^ Each of these 5 methods incorporates unique analytical assumptions and approaches, enriching the causal estimation from various perspectives.^[26]^ Furthermore, pleiotropy and heterogeneity tests were run. As IVs, we employed MR-Egger regression to examine the possible pleiotropic effects of the chosen SNPs.^[27]^ Heterogeneity was assessed using the Cochran *Q* test; *P*-values higher than .05 indicated that there was no significant heterogeneity. To ensure that no single SNP substantially associated with the exposure would unduly impact the causal effect estimate, a leave-one-out analysis was also conducted. Subsequently, a multivariable Mendelian randomization analysis was conducted on the chosen exposures and outcomes to determine the Comprehensive effect of multiple exposures on disease risk.

## 3. Results

### 3.1. MR analysis

**Figure 4.**
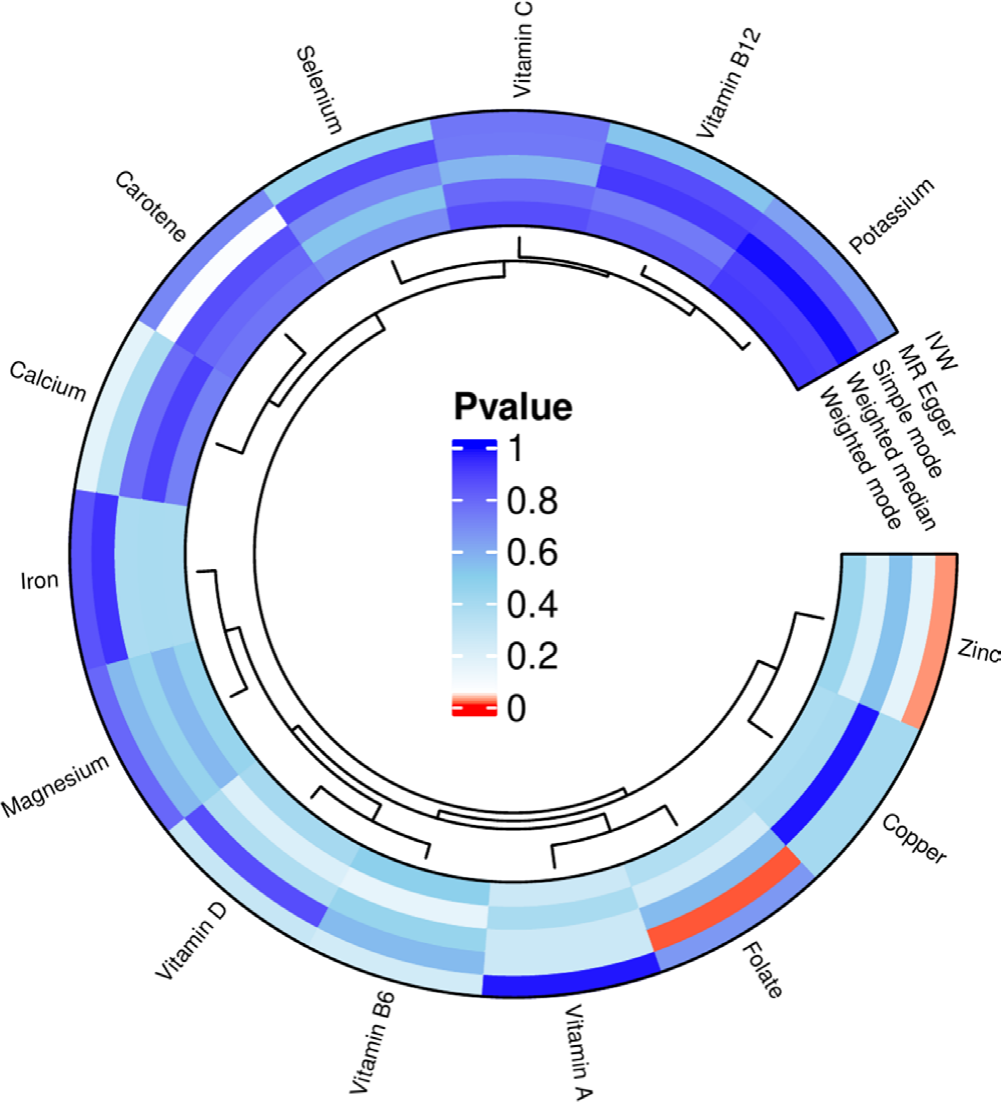
MR analysis results for exposures (copper, calcium, iron, magnesium, potassium, selenium, zinc, carotene, folate, vitamins A, B_6_, B_12_, C, and D) and outcome (CH), utilizing methods including IVW, weighted median, MR-Egger, simple mode, and weighted mode. CH = chronic hepatitis, IVW = inverse variance weighting, MR = Mendelian randomization.

The MR analysis involved 14 micronutrients – copper, selenium, magnesium, zinc, calcium, iron, potassium, carotene, folate, and vitamins A, B_6_, B_12_, C, and D – as exposures, with CH serving as the outcome. Following adjustments, a random-effects IVW analysis confirmed significant causal associations for zinc (*P* = .022, odds ratio (OR) = 0.806, 95% confidence interval (CI) = 0.670–0.970) with CH. The associations for copper, selenium, magnesium, calcium, iron, potassium, carotene, folate, and vitamins A, B_6_, B_12_, C, and D (IVW *P* > .05) were not statistically significant. Despite the MR-Egger analysis for folate (*P* = .009, OR = 0.0536, 95% CI = 0.009–0.320) indicating no heterogeneity (*P* > .05), it presented contradictory findings compared to the IVW method. These results are illustrated in Figure 4 and Supplementary File 1, Supplemental Digital Content, https://links.lww.com/MD/R228.

**Figure 5.**
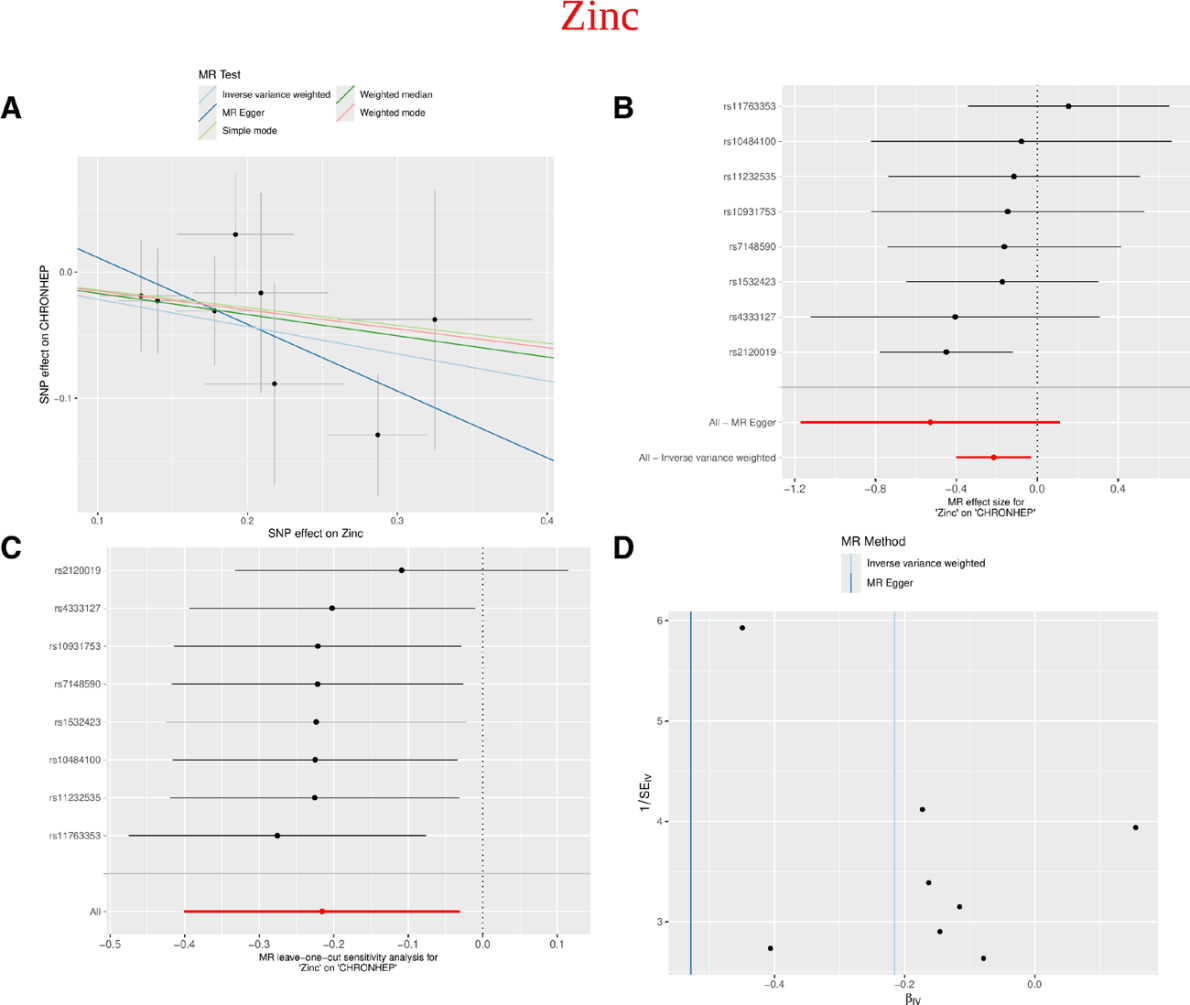
MR analysis of the causal relationship between zinc and CH. (A) Scatter plot illustrating the causal effect of zinc on CH: the slope indicates the effect’s magnitude, with black points representing the genetic instruments used in the primary MR analysis. (B) Forest plot visualizing the causal effect of each SNP on CH risk. (C) Leave-one-out plot analyzing the stability of the causal relationship between zinc and CH. (D) Funnel plot displaying SNP heterogeneity. CH = chronic hepatitis, MR = Mendelian randomization, SNP = single nucleotide polymorphism

After filtering micronutrients linked to the illness using the R package, the analysis revealed that zinc had a pleiotropy of *P* = .357 (*P* > .05), suggesting that the analysis was not affected by horizontal pleiotropy (Supplementary File 2, Supplemental Digital Content, https://links.lww.com/MD/R228). Zinc was identified as the micronutrient linked to CH by MR analysis (Supplementary File 3, Supplemental Digital Content, https://links.lww.com/MD/R228), and the IVW analysis identified zinc (*P* = .022, OR = 0.806, 95% CI = 0.670–0.970). Meanwhile, Tests for pleiotropy (Supplementary File 4, Supplemental Digital Content, https://links.lww.com/MD/R228) and heterogeneity (Supplementary File 5, Supplemental Digital Content, https://links.lww.com/MD/R228) revealed *P*-values higher than .05.Outlier detection results (Supplementary File 6, Supplemental Digital Content, https://links.lww.com/MD/R228) showed that all combinations used for outlier detection had *P*-values > .05, and the individual SNP outlier test (Supplementary File 7, Supplemental Digital Content, https://links.lww.com/MD/R228) did not find any SNP outliers. Scatter plots showed consistent results across all 5 methods for SNPs relating to the exposure and outcome. Leave-one-out sensitivity analysis confirmed that the exclusion of any single SNP did not alter the findings. Additionally, the funnel plots in Figure 5 satisfy the requirements.

### 3.2. Multivariable MR analysis

**Figure 6.**
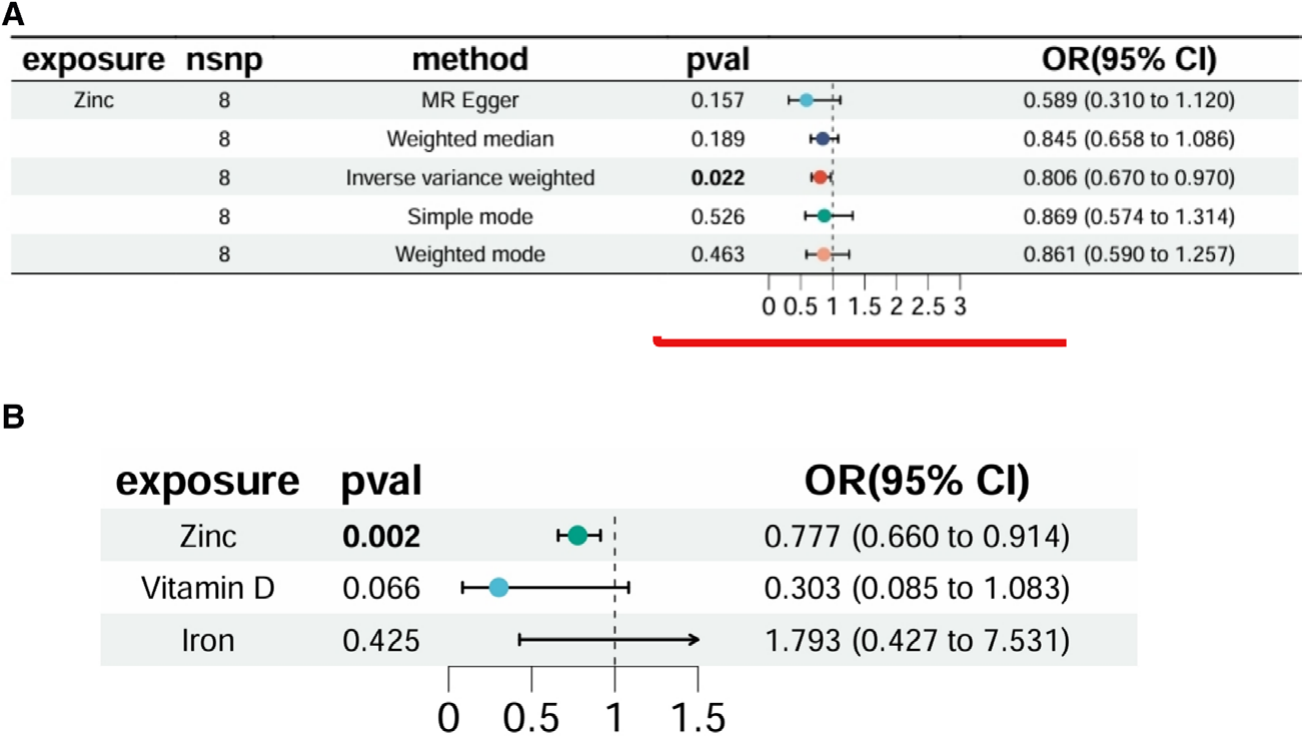
Forest plots. (A) Forest plot of zinc MR analysis with inverse variance weighting (IVW), weighted median, MR-Egger, simple mode, and weighted mode. (B) Forest plot of inverse variance weighting MR analysis for zinc, vitamin D, and iron. IVW = inverse variance weighting, MR = Mendelian randomization.

Vitamin D and iron, along with zinc, were analyzed in the multivariable Mendelian randomization framework (Supplementary File 8, Supplemental Digital Content, https://links.lww.com/MD/R228). The analysis showed marginally statistically significant independent causal effects for vitamin D (*P* = .066, OR = 0.303, 95% CI = 0.0846–1.083) and no statistically significant causal effects of iron (*P* = .425, OR = 1.793, 95% CI = 0.427–7.531) on CH. Zinc, however, demonstrated an independent protective effect against CH (*P* = .002, OR = 0.777, 95% CI = 0.660–0.914). Tests for heterogeneity and pleiotropy yielded *Q* values with *P* > .05, confirming the absence of significant heterogeneity or pleiotropy (Supplementary File 9, Supplemental Digital Content, https://links.lww.com/MD/R228). Comparative analysis of forest plots for zinc alone and in combination with vitamin D and iron is presented in Figure 6, affirming zinc’s causal relationship with CH, thus establishing its protective role against CH.

## 4. Discussion

In 1994, the World Congresses of Gastroenterology and Hepatology, held in Cancun, Mexico, and Los Angeles, USA, established the foundational definition of CH. This consensus defined CH as liver inflammation with histopathological changes persisting for over 6 months. Additionally, the meeting emphasized the importance of incorporating etiology into the classification of CH, recognizing the diverse causes underlying its pathogenesis.

Chronic viral infections, particularly HBV and HCV, represent the predominant cause of CH today.^[28-30]^ The clinical progression of CH is closely intertwined with micronutrient status, which may modulate disease outcomes. This Mendelian randomization (MR) study aimed to elucidate potential causal associations between CH and 14 micronutrients: selenium, copper, magnesium, vitamins A, B_6_, B_12_, C, and D, zinc, folate, and carotene. Our findings revealed a significant protective effect of zinc against CH, highlighting its potential role in mitigating disease progression.

Zinc, the second most abundant trace element in the human body,^[31]^ maintains physiological levels of approximately 1.4 to 2.3 g, with 60% distributed in skeletal muscle and 30% in bone.^[11,32]^ In the context of HBV infection, T cells, particularly CD8+ T cells, play a critical role in disease pathophysiology. Research demonstrates that depletion of CD8+ T cells significantly prolongs HBV infection and delays viral clearance during the early acute phase.^[33,34]^ Zinc facilitates T-cell activation by promoting the production of lymphocyte protein tyrosine kinase and protein kinase C.^[35-37]^ Additionally, it regulates the development, activation, and differentiation of T cells.^[38]^

The liver plays a pivotal role in maintaining zinc homeostasis, which is disrupted in CH due to various etiologies, often resulting in zinc deficiency.^[39]^ In advanced ALD, patients exhibit reduced serum and hepatic zinc concentrations alongside elevated urinary zinc excretion.^[40,41]^ Studies indicate that ethanol consumption increases urinary zinc loss while impairing intestinal zinc absorption, potentially due to enhanced intestinal permeability.^[42-44]^ Furthermore, ethanol exposure downregulates hepatic zinc transporters ZIP5 and ZIP14 and suppresses zinc-binding proteins by reducing hepatocyte nuclear factor-4α levels, collectively contributing to disrupted hepatic zinc homeostasis.^[45]^ In ALD, zinc enhances cellular antioxidant capacity, restores hepatocyte nuclear factor-4α and peroxisome proliferator-activated receptor-α activity, and promotes hepatic fatty acid β-oxidation and lipid secretion.^[46]^ More broadly, zinc acts as a hepatoprotective agent by modulating lipid metabolism, bolstering antioxidant defenses, regulating cell proliferation, and inhibiting apoptosis in hepatic tissues.^[47]^ In HCV infection, persistent viral activity induces mitochondrial OS, leading to metabolic disturbances such as insulin resistance and hepatic steatosis. Zinc exerts a protective role by mitigating OS, apoptosis, and inflammation.^[48-51]^ In non-AFLD, lower serum levels of zinc-alpha-2-glycoprotein are associated with increased severity of hepatic steatosis.^[52]^ In nonalcoholic steatohepatitis resulting from non-AFLD, zinc deficiency exacerbates mitochondrial OS, contributing to iron overload, insulin resistance, and hepatic steatosis.^[53]^

In clinical settings, the zinc-carnosine complex, polaprezinc, has shown promise in mitigating liver fibrosis in nonalcoholic steatohepatitis by reducing inflammation and lipid peroxidation.^[54,55]^ Additionally, polaprezinc promotes the reversal of liver fibrosis by inactivating hepatic stellate cells.^[56]^ Animal studies further demonstrate that Polaprezinc prevents liver fibrosis by suppressing hepatic stellate cell activation, reducing OS, and decreasing levels of hepatic hydroxyproline, tissue inhibitor of metalloproteinase-1, and transforming growth factor-β1.^[57]^ In the context of HCV infection, zinc supplementation has been associated with a reduced incidence of HCC, likely due to elevated serum zinc levels.^[58]^ Moreover, in refractory chronic hepatitis C (CHC), zinc supplementation enhances the therapeutic response to interferon therapy.^[59,60]^ Collectively, these findings reinforce zinc’s role as a protective factor in CH, highlighting its potential in therapeutic interventions.

Our research offers fresh insights into CH treatment, albeit with notable limitations. Our results may not be as generalizable to other racial or ethnic groups or geographical areas because the GWAS datasets we used were taken from European populations. Furthermore, the data were exclusively categorized by European ancestry, neglecting to consider age, gender, or dietary influences, which could introduce bias into the findings. Furthermore, owing to a limited quantity of SNPs available for MR analysis, we modified the genome-wide significance threshold to 5 × 10^−6^, which may influence the dependability of our results. Ultimately, although we conducted tests for pleiotropy and consistency, we cannot ensure that the results were free from the influence of unidentified pleiotropic variants. Future investigations should focus on enhancing the design by incorporating larger sample sizes, a broader range of populations, and more rigorous screening criteria.

## 5. Conclusion

In summary, our research confirms zinc as a protective factor for CH, offering new perspectives for the clinical understanding and treatment of CH. However, we have not determined the relationship between other micronutrients and CH, nor have we explored the potential synergistic effects between zinc and other nutrients. Sincerely hope that future researchers can adopt other research methods to further investigate these aspects.

## Acknowledgments

Thank you to everyone who contributed to this study.

## Author contributions

**Conceptualization:** Yong Qi.

**Data curation:** ZhengHao Zhang, TianYang Chen.

**Formal analysis:** ZhengHao Zhang, TianYang Chen.

**Methodology:** Yong Qi.

**Software:** ZhengHao Zhang, TianYang Chen.

**Supervision:** Yong Qi.

**Validation:** ZhengHao Zhang, TianYang Chen, Yong Qi.

**Writing – original draft:** ZhengHao Zhang, TianYang Chen, Yong Qi.

**Writing – review & editing:** ZhengHao Zhang, TianYang Chen.

## Supplementary Material



## References

[1] Suriawinata AA Thung SN. Acute and chronic hepatitis. Semin Diagn Pathol. 2006;23:132–48. 17355087 10.1053/j.semdp.2006.11.001

[2] World Health O. Global Progress Report on HIV, Viral Hepatitis and Sexually Transmitted Infections, 2021: Accountability for the Global Health Sector Strategies 2016–2021: Actions for Impact: Web Annex 1: Key Data at a Glance. World Health Organization; 2021.

[3] Guidotti LG Chisari FV. Immunobiology and pathogenesis of viral hepatitis. Annu Rev Pathol. 2006;1:23–61. 18039107 10.1146/annurev.pathol.1.110304.100230

[4] Rehermann B. Pathogenesis of chronic viral hepatitis: differential roles of T cells and NK cells. Nat Med. 2013;19:859–68. 23836236 10.1038/nm.3251 PMC4482132

[5] Fan R Papatheodoridis G Sun J. aMAP risk score predicts hepatocellular carcinoma development in patients with chronic hepatitis. J Hepatol. 2020;73:1368–78. 32707225 10.1016/j.jhep.2020.07.025

[6] Sun K Zhao JV Nelson EAS Wong VWS Lam HSHS Hui LL. Iron status and non-alcoholic fatty liver disease: a Mendelian randomization study. Nutrition. 2024;118:112295. 38103266 10.1016/j.nut.2023.112295

[7] Zoroddu MA Aaseth J Crisponi G Medici S Peana M Nurchi VM. The essential metals for humans: a brief overview. J Inorg Biochem. 2019;195:120–9. 30939379 10.1016/j.jinorgbio.2019.03.013

[8] Luft FC. Blood and iron. J Mol Med (Berl). 2015;93:469–71. 25877861 10.1007/s00109-015-1284-0

[9] Bonkovsky HL Banner BF Rothman AL. Iron and chronic viral hepatitis. Hepatology. 1997;25:759–68. 9049232 10.1002/hep.510250345

[10] Kaito M. Molecular mechanism of iron metabolism and overload in chronic hepatitis C. J Gastroenterol. 2007;42:96–9. 17323001 10.1007/s00535-006-1983-y

[11] Kumar M Abbas Z Gazder DP Nazir S Maqbool S Qadeer MA. Deficiencies of micronutrients in potential liver transplant candidates: a cross-sectional study. Euroasian J Hepato Gastroenterol. 2025;15:24–8. 10.5005/jp-journals-10018-1466 PMC12288596 40718614

[12] Kato J Kobune M Nakamura T. Normalization of elevated hepatic 8-hydroxy-2’-deoxyguanosine levels in chronic hepatitis C patients by phlebotomy and low iron diet. Cancer Res. 2001;61:8697–702. 11751387

[13] He R Wei Y Peng Z. α-Ketoglutarate alleviates osteoarthritis by inhibiting ferroptosis via the ETV4/SLC7A11/GPX4 signaling pathway. Cell Molecular Biol Lett. 2024;29:88. 10.1186/s11658-024-00605-6 PMC11177415 38877424

[14] Rahman AH Branch AD. Vitamin D for your patients with chronic hepatitis C? J Hepatol. 2013;58:184–9. 22871501 10.1016/j.jhep.2012.07.026

[15] Jeffery L Raza K Filer A Sansom D. 25-hydroxyvitamin D3 conversion by dendritic cells and T cells drives 1,25-dihydroxyvitamin D3 mediated anti-inflammatory CD4+ T cell responses. Ann Rheum Dis. 2011;70:A45.

[16] Dai J Song J Chen X. 1,25(OH)(2)D(3)-treated mouse bone marrow-derived dendritic cells alleviate autoimmune hepatitis in mice by improving TFR/TFH imbalance. Immunopharmacol Immunotoxicol. 2025;47:59–67. 39604017 10.1080/08923973.2024.2435314

[17] Gutierrez JA Parikh N Branch AD. Classical and emerging roles of vitamin D in hepatitis C virus infection. Semin Liver Dis. 2011;31:387–98. 22189978 10.1055/s-0031-1297927 PMC4107414

[18] Antonow DR McClain CJ. Nutritional support in alcoholic liver disease. JPEN J Parenter Enteral Nutr. 1985;9:566–7. 4046169 10.1177/0148607185009005566

[19] Larsson SC Butterworth AS Burgess S. Mendelian randomization for cardiovascular diseases: principles and applications. Eur Heart J. 2023;44:4913–24. 37935836 10.1093/eurheartj/ehad736 PMC10719501

[20] Chen T Gu Y Zhang Z Chen Z Zhang J Leng X. Association between copper and Achilles tendon disease: a two-sample Mendelian randomization study. Front Nutr. 2024;11:1505636. 39606572 10.3389/fnut.2024.1505636 PMC11598432

[21] Liang Y Luo S Bell S. Do iron homeostasis biomarkers mediate the associations of liability to type 2 diabetes and glycemic traits in liver steatosis and cirrhosis: a two-step Mendelian randomization study. BMC Med. 2024;22:270. 38926684 10.1186/s12916-024-03486-w PMC11210020

[22] Zhou B-G Xia J-L Jiang X Ding Y-B She Q. Non-alcoholic fatty liver disease and gestational diabetes mellitus: a bidirectional two-sample mendelian randomization study. BMC Endocr Disord. 2024;24:40. 38504196 10.1186/s12902-024-01569-6 PMC10953072

[23] Skrivankova VW Richmond RC Woolf BAR. Strengthening the reporting of observational studies in epidemiology using mendelian randomization: the STROBE-MR statement. JAMA. 2021;326:1614–21. 34698778 10.1001/jama.2021.18236

[24] Xia T Du M Li H. Association between liver MRI proton density fat fraction and liver disease risk. Radiology. 2023;309:e231007. 37874242 10.1148/radiol.231007

[25] Tuo L Yan L-T Liu Y Yang X-X. Type 1 diabetes mellitus and non-alcoholic fatty liver disease: a two-sample Mendelian randomization study. Front Endocrinol (Lausanne). 2024;15:1315046. 38681765 10.3389/fendo.2024.1315046 PMC11045944

[26] Liu Y Wang X You M Zheng M Yu M Leng X. Association between vitamin B6 levels and rheumatoid arthritis: a two-sample Mendelian randomization study. Front Nutr. 2024;11:1442214. 39464681 10.3389/fnut.2024.1442214 PMC11502391

[27] Burgess S Thompson SG. Interpreting findings from Mendelian randomization using the MR-Egger method. Eur J Epidemiol. 2017;32:377–89. 28527048 10.1007/s10654-017-0255-x PMC5506233

[28] Bradbear RA. Chronic hepatitis: a review. J R Soc Med. 1985;78:391–6. 3921709 10.1177/014107688507800509 PMC1289721

[29] Maddrey WC. Chronic hepatitis. Dis Mon. 1993;39:53–125. 8472614

[30] Brunt EM. Grading and staging the histopathological lesions of chronic hepatitis: the Knodell histology activity index and beyond. Hepatology. 2000;31:241–6. 10613753 10.1002/hep.510310136

[31] Duan M Li T Liu B. Zinc nutrition and dietary zinc supplements. Crit Rev Food Sci Nutr. 2023;63:1277–92. 34382897 10.1080/10408398.2021.1963664

[32] Maughan RJ. Role of micronutrients in sport and physical activity. Br Med Bull. 1999;55:683–90. 10746356 10.1258/0007142991902556

[33] Thimme R Wieland S Steiger C. CD8(+) T cells mediate viral clearance and disease pathogenesis during acute hepatitis B virus infection. J Virol. 2003;77:68–76. 12477811 10.1128/JVI.77.1.68-76.2003 PMC140637

[34] Kocyła A Krężel A. Zinc-mediated dynamics of CD4/CD8α co-receptors and Lck kinase: implications for zinc homeostasis, immune response, and biotechnological innovations. Metallomics. 2025;17:mfaf018. 40504517 10.1093/mtomcs/mfaf018 PMC12198760

[35] Korichneva I Hoyos B Chua R Levi E Hammerling U. Zinc release from protein kinase C as the common event during activation by lipid second messenger or reactive oxygen. J Biol Chem. 2002;277:44327–31. 12213816 10.1074/jbc.M205634200

[36] Pernelle JJ Creuzet C Loeb J Gacon G. Phosphorylation of the lymphoid cell kinase p56lck is stimulated by micromolar concentrations of Zn2+. FEBS Lett. 1991;281:278–82. 2015905 10.1016/0014-5793(91)80411-u

[37] Yang F Smith MJ Siow RCM Aarsland D Maret W Mann GE. Interactions between zinc and NRF2 in vascular redox signalling. Biochem Soc Trans. 2024;52:269–78. 38372426 10.1042/BST20230490 PMC10903478

[38] Lan L Feng Z Liu X Zhang B. The roles of essential trace elements in T cell biology. J Cell Mol Med. 2024;28:e18390. 38801402 10.1111/jcmm.18390 PMC11129730

[39] Kohgo Y Ikuta K Ohtake T Torimoto Y Kato J. Iron overload and cofactors with special reference to alcohol, hepatitis C virus infection and steatosis/insulin resistance. World J Gastroenterol. 2007;13:4699–706. 17729391 10.3748/wjg.v13.i35.4699 PMC4611191

[40] Kahn AM Helwig HL Redeker AG Reynolds TB. Urine and serum zinc abnormalities in disease of the liver. Am J Clin Pathol. 1965;44:426–35. 5839914 10.1093/ajcp/44.4.426

[41] Parisse S Andreani G Mischitelli M. Differences in tissue copper and zinc content between normal livers and those with cirrhosis with or without hepatocellular carcinoma. Int J Mol Sci . 2025;26:6571. 40724821 10.3390/ijms26146571 PMC12294540

[42] Kang YJ Zhou Z. Zinc prevention and treatment of alcoholic liver disease. Mol Aspects Med. 2005;26:391–404. 16099027 10.1016/j.mam.2005.07.002

[43] Barve S Chen S-Y Kirpich I Watson WH McClain C. Development, prevention, and treatment of alcohol-induced organ injury: the role of nutrition. Alcohol Res. 2017;38:289–302. 28988580 10.35946/arcr.v38.2.11 PMC5513692

[44] Kang YF Zhao JY Liu JR. Zinc sulfate improves insulin resistance, oxidative stress and apoptosis in liver tissues of PCOS rats through the NF-κB pathway. Front Endocrinol (Lausanne). 2025;16:1569866. 40547527 10.3389/fendo.2025.1569866 PMC12178883

[45] Sun Q Li Q Zhong W. Dysregulation of hepatic zinc transporters in a mouse model of alcoholic liver disease. Am J Physiol Gastrointest Liver Physiol. 2014;307:G313–22. 24924749 10.1152/ajpgi.00081.2014 PMC4121635

[46] Zhou Z. Zinc and alcoholic liver disease. Dig Dis. 2011;28:745–50. 10.1159/000324282 PMC7065391 21525759

[47] Iritani S Kawamura Y Muraishi N. The useful predictors of zinc deficiency for the management of chronic liver disease. J Gastroenterol. 2022;57:322–32. 35233650 10.1007/s00535-022-01852-0

[48] Prasad AS Bao B Beck FWJ Kucuk O Sarkar FH. Antioxidant effect of zinc in humans. Free Radic Biol Med. 2004;37:1182–90. 15451058 10.1016/j.freeradbiomed.2004.07.007

[49] Ko W-S Guo C-H Yeh M-S. Blood micronutrient, oxidative stress, and viral load in patients with chronic hepatitis C. World J Gastroenterol. 2005;11:4697–702. 16094713 10.3748/wjg.v11.i30.4697 PMC4615414

[50] Tantai X Wen Z Tuo S. Associations of serum vitamin D with sarcopenia in patients with chronic liver disease: a population-based cross-sectional study. Calcif Tissue Int. 2025;116:69. 40325227 10.1007/s00223-025-01376-8

[51] Gao T Chen T Ai C. The causal relationship between zinc and osteoarthritis: a two-sample mendelian randomization study. Biol Trace Elem Res. 2025;203:5890–900. 40195255 10.1007/s12011-025-04611-3 PMC12602555

[52] Qi X-Y Li J-Y Wang Y-D. Association of serum zinc-α2-glycoprotein with non-alcoholic fatty liver disease. Chin Med J (Engl). 2020;133:1882–3. 32590463 10.1097/CM9.0000000000000873 PMC7470012

[53] Himoto T Masaki T. Associations between zinc deficiency and metabolic abnormalities in patients with chronic liver disease. Nutrients. 2018;10:88. 29342898 10.3390/nu10010088 PMC5793316

[54] Sugino H Kumagai N Watanabe S. Polaprezinc attenuates liver fibrosis in a mouse model of non-alcoholic steatohepatitis. J Gastroenterol Hepatol. 2008;23:1909–16. 18422963 10.1111/j.1440-1746.2008.05393.x

[55] Dungubat E Fujikura K Kuroda M Fukusato T Takahashi Y. Food nutrients and bioactive compounds for managing metabolic dysfunction-associated steatotic liver disease: a comprehensive review. Nutrients. 2025;17:2211. 40647314 10.3390/nu17132211 PMC12251994

[56] Ye J Zhang Z Zhu L. Polaprezinc inhibits liver fibrosis and proliferation in hepatocellular carcinoma. Mol Med Rep. 2017;16:5523–8. 28849143 10.3892/mmr.2017.7262

[57] Kono T Asama T Chisato N. Polaprezinc prevents ongoing thioacetamide-induced liver fibrosis in rats. Life Sci. 2012;90:122–30. 22100444 10.1016/j.lfs.2011.10.022

[58] Matsumura H Nirei K Nakamura H. Zinc supplementation therapy improves the outcome of patients with chronic hepatitis C. J Clin Biochem Nutr. 2012;51:178–84. 23170044 10.3164/jcbn.12-11 PMC3491241

[59] Takagi H Nagamine T Abe T. Zinc supplementation enhances the response to interferon therapy in patients with chronic hepatitis C. J Viral Hepat. 2001;8:367–71. 11555194 10.1046/j.1365-2893.2001.00311.x

[60] El-Haggar SM Attalla DS Elhelbawy M El-Afify DR. A randomized clinical study to evaluate the possible antifibrotic effect of zinc sulfate in chronic HCV patient receiving direct-acting anti-viral therapy. Inflammopharmacology. 2025;33:329–39. 39789197 10.1007/s10787-024-01628-3 PMC11799079

